# S250. RELATION BETWEEN PSYCHOPATHOLOGY AND QUALITY OF LIFE IN SCHIZOPHRENIA PATIENTS BEFORE AND AFTER FIRST ANTIPSYCHOTIC TREATMENT

**DOI:** 10.1093/schbul/sby018.1037

**Published:** 2018-04-01

**Authors:** Gitte Andersen, Helle Schæbel, Sanne Wulff, Birte Glenthøj, Mette Ødegaard Nielsen

**Affiliations:** Center for Neuropsychiatric Schizophrenia Research, CNSR, and Center for Clinical Intervention and Neuropsychiatric Schizophrenia Research, CINS, Mental Health Centre Glostrup, University of Copenhagen

## Abstract

**Background:**

It is common knowledge that antipsychotic treatment improves the symptomatology in schizophrenia, especially for the psychotic and general symptoms. It is also a fact that patients with schizophrenia often report a reduced quality of life compared to healthy controls. In this study we aim at examining the relation between self-reported quality of life (QLS), psychopathological symptoms and level of function before and after antipsychotic treatment. We hypothesize that there will be a correlation between QLS and severity of symptoms before treatment. Further we expect an improvement in QLS after treatment and that this improvement will correlate with improvement in symptomatology.

**Methods:**

As a part of a large multimodal study on antipsychotic naïve patients with schizophrenia, 69 patients were recruited. Their psychopathology was measured with the Positive and Negative Syndrome Scale (PANSS), level of function was estimated using Global Assessment of Function (GAF), and QLS was reported by answering a questionnaire. Patients were treated with individual doses of Amisulpride for six weeks, after which they were reexamined.

The questionnaire regarding QLS counts 21 questions, divided into four domains: Self and present life (i.e. “how satisfied are you with your present life”), social relations (“how satisfied are you with your current social life”), Living situation (“how much do you like the place you live”) and Work situation (“How satisfied are you with the work you do”). Higher scores indicate higher satisfaction within the domain. Since the follow up period was only 6 weeks, we focused on self and present life (SPL) and social relations (SR), as we did not expect the living and work situation to change significantly within this period.

**Results:**

Baseline data were available on 48 patients, mean age 25 years (6.1), 31 males (65%).

Their PANSS total score was 84 (16.0), GAF was 41(9.4), SPL-score was 13 (5.3) SR-score 10(5.3). For SPL as well as SR, there was a negative correlation with PANSS-total, PANSS-negative and PANSS- general (p-values<0.007). Follow-up data were available on 33 patients, mean age 25 years (6.6), 19 males (58%). They received 273 (163.3) mg Amisuplride. PANSS total was 68 (14.4) and GAF was 53 (15.7), SPL-score was 14 (3.9) SR-score 11(4.6). Paired T-test showed a significant improvement in PANSS total, PANSS positive, PANSS general and GAF (all p-values<0.001). There was also an improvement in SR (p=0.003), but no significant improvement in SPL and PANSS negative score (p=0.12 and p=0.5).

There were no correlations between neither of the QLS scores and any psychopathology scores at follow up. Likewise, there was no correlation between change in QLS scores and change in psychopathology. However, there was a negative correlation with change in SPL and medication dose (p=0.009)

**Discussion:**

In this study, we found that antipsychotic naïve patients with most severe symptoms had the lowest self-reported QLS. This relation was only observed for negative and general symptoms, but not for positive symptoms or GAF score. As expected there was a treatment induced improvement in positive and general symptoms as well as GAF score. Likewise, patients improved on QLS, but only on SR and not in the overall measure of SPL. This may partly be because antipsychotic medication primarily improves positive symptoms, which were not correlated with QLS. Additionally, there was even a negative correlation with medication dose, indicating that patients with higher doses had the least improvement I SPL score. The results indicate that there is not a simple relationship between antipsycotic induced improvement in psychopathology and selfreported QLS. High doses of medication may even reduce QLS.

